# 宣威肺癌分子流行病学研究：煤种、基因型与肺癌风险

**DOI:** 10.3779/j.issn.1009-3419.2015.01.03

**Published:** 2015-01-20

**Authors:** 继华 李, 俊 何, 锐 唐, Wei HU, Wei LAN, 兴舟 何, 云 李, 云生 张

**Affiliations:** 1 655000 曲靖，曲靖市疾病预防控制中心 Qujing Centers for Disease Control and Prevention, Qujing 655000, China; 2 655000 曲靖，曲靖市卫生局 Qujing Municipal Bureau of Health, Qujing 655000, China; 3 20892 Bethesda, National Cancer Institute National Cancer Institute, National Institutes of Health, Bethesda 20892, MD, USA; 4 100050 北京，中国疾病预防控制中心 Chinese Centers for Disease Control and Prevention, Beijing 100050, China

**Keywords:** 肺肿瘤, 烟煤, GSTT1, AKR1C3, OGG1, 宣威, Lung neoplasms, Smoky coal, GST, AKR1C3, OGG, Xuanwei county

## Abstract

**背景与目的:**

已有的研究证明：宣威是我国农村肺癌死亡率最高的地区，肺癌危险与室内燃烧烟煤产生的多环芳烃（polycyclic aromatic hydrocarbons, PAHs）有关，肺癌发病具有明显的地域性差别和家族聚集性。本研究从分子流行病学角度探索宣威肺癌风险的危险因素及与发病机理有关的基因型和燃煤类型。

**方法:**

运用两个基于人群的病例对照研究，开展问卷调查，同时采集口腔细胞和痰等生物样品，提取DNA。应用PCR法检测GST超家族、AKR超家族和OGG1等基因型。通过*Logistic*回归分析煤种、基因型与肺癌风险的关联。

**结果:**

与燃用无烟煤或木柴的研究对象相比，家用来宾烟煤的肺癌风险比数比（odds ratio, OR）值高达24.8，其次为龙潭（OR=11.6）、宝山（OR=6.0）、龙场（OR=4.1）、羊场（OR=3.8）等；燃用同种烟煤的男女肺癌风险相似。对于GSTM1缺失、AKR1C3（Ex1-70C > G）、OGG1（Ex6-315C > G）基因型，肺癌风险明显增高，且具统计学意义[OR（95%CI）分别为2.3（1.3-4.2）、1.8（1.0-3.5）和1.9（1.1-3.3）]。与家用烟煤量小且具GSTM1阳性的研究对象相比，用量大且GSTM1缺失的研究对象肺癌风险更高，女性OR为4.9（1.3-18.2），男性OR为2.7（1.0-7.4）。但对于AKR1C3（Ex1-70C > G）和OGG1（Ex6-315C > G），仅观察到女性肺癌风险的明显增高，OR分别为12.9（2.2-107.8）和5.7（1.1-34.2）。

**结论:**

宣威不同煤种的肺癌风险有很大差异，但暴露于相同煤种的男性与女性肺癌风险相似。GSTM1缺失基因型与肺癌风险增高有关联。AKR1C3与OGG1这2种基因型与肺癌风险有关联，且在烟煤使用量高的女性中肺癌风险更高。

## Abstract (en)

**Background and objective:**

It has been proven that the lung cancer mortality rate in Xuanwei County, China was among the highest in the country and has been associated with exposure to indoor smoky coal emissions that contain high levels of polycyclic aromatic hydrocarbons. This risk may be modified by variation in genetic polymorphisms and coal subtypes. Our objective was to use molecular epidemiological techniques to investigate the relationship among genetic polymorphisms, coal subtype and lung cancer risk in Xuanwei County.

**Methods:**

On the basis of two population-based case-control studies in residents of Xuanwei County, China, questionnaires covering demographic information, smoking history, family and personal medical history, and information on other variables were administered and buccal cells and sputum samples were collected separately from each subject enrolled to extract DNA. GST superfamily, AKR1C3 superfamily, OGG1 superfamily and other genotype were scanned by useing PCR method. ORs and 95%CIs were used to estimate the association between genotypes, coal subtypes and lung cancer risk factors by conditional *Logistic* regression using Statistical Analysis Software.

**Results:**

Compared with subjects who using smokeless coal or wood, smoky coal use was statistically significantly associated with lung cancer risk (OR=7.7, 95%CI: 4.5-13.3). There was marked heterogeneity in risk estimates for specific subtypes of smoky coal. Estimates were highest for coal from the Laibin (OR=24.8), Longtan (OR=11.6) and Baoshan (OR=6.0) coal types, and lower for coal from other types; the risk within the same subtype of coal in male and female were similar. The GSTM1-null genotype, the AKR1C3 (Ex1-70C > G), OGG1 (Ex6-315C > G) genotypes were closely associated with increased risk of lung cancer in Xuanwei County, and their odds ratios (95%CI) were 2.3 (1.3-4.2), 1.8 (1.0-3.5) and 1.9 (1.1-3.3), respectively. Compared to subjects who with GSTM1-positive and used less than 130 tons of smoky coal during their lifetime, higher risks were closely associated with GSTM1-null and heavier users (≥130 tons), with the OR was 4.9 (95%CI: 1.3-18.2) and 2.7 (95%CI: 1.0-7.4) for female and male, respectively. However, higher risks were only found within female for AKR1C3 (Ex1-70C > G) and OGG1 (Ex6-315C > G), with OR (95%CI)=12.9 (2.2-107.8) and 5.7 (1.1-34.2), respectively.

**Conclusion:**

Lung cancer risks varied among coal subtypes; however, risks were similar between men and women exposed to the same type of coal. The GSTM1-null genotype may enhance susceptibility to air pollution from indoor smoky coal combustion emissions. AKR1C3 and OGG1 genotypes were significantly associated with higher risk of lung cancer, especially among heavily exposed women.

云南宣威是滇东黔西晚二叠世聚煤区主要产煤地之一，数百年来当地居民就近取煤作为做饭、取暖的主要生活燃料。20世纪70年代全国死因调查发现宣威县是我国肺癌死亡率最高地区之一，男、女性肺癌死亡率分别是全国平均水平的4倍和8倍^[1, 2]^。一般而言，男性肺癌死亡率通常高于女性，这可能与男性吸烟有关。当地女性居民基本不吸烟，但肺癌死亡率却与男性相差不大（1973年-1979年男女肺癌死亡率分别为27.7/10万和25.3/10万^[3, 4]^）。早期研究^[2-5]^表明，居民室内使用无烟囱火塘燃烧烟煤产生很高浓度的空气颗粒物和苯并a芘（benzo[a]pyrene, BaP）以及其他的有机化合物^[1, 4]^，室内燃煤与肺癌之间存在较高的关联。毒理学研究^[6, 7]^也显示宣威的烟煤燃烧产物比无烟煤和木柴燃烧产物更具致癌致突变毒性。同时，研究^[3, 4]^还发现虽然居民生活同样使用烟煤，但由于煤源不同，不同地区（乡镇）肺癌死亡率相差数倍至数十倍之多；即便是同一地区不同家族、同家族中有血缘关系的一级亲属与配偶家系间肺癌风险差别也很大，肺癌流行具有明显的家族聚集性^[4, 8]^，在肺癌的发生过程中基因变异可能起到了重要作用^[9, 10]^。

多环芳烃（polycyclic aromatic hydrocarbons, PAHs）进入人体后，大部分经混合功能氧化酶代谢生成多种中间产物或终产物，其中一些代谢产物与DNA共价形成PAH-DNA加合物，引起DNA损伤，诱导基因突变，甚至诱发肿瘤形成。多态性谷胱甘肽-S-转移酶（glutathione S-transferase, GST）基因超家族控制了内源性和外源物与谷胱甘肽的结合酶，这些基因在解毒和偶然性外源物激活过程中可能发挥重要作用。正常或增强的GST酶活性会通过这种结合限制亲电性致癌物，从而保护敏感组织发生细胞DNA突变。在*GSTM1*和*GSTT1*基因中纯合子缺失的载体会分别呈现出GSTM1和GSTT1酶活性失活，而GSTM1对PAHs代谢产物有解毒作用，GSTT1对其他可能的致癌物质（例如卤代甲烷、环氧乙烷）有解毒作用^[11-13]^。另外，PAHs至少通过三种代谢途径产生具有基因毒性的中间产物。这三种途径都会发生碱基G和T的颠换。其中一种途径涉及到二氢二醇脱氢酶，它是醛酮还原酶（aldo-keto reductase, AKR）超家族中的一员，而且此途径会形成DNA加合物和氧自由基（reactive oxygen species, ROS）的中间产物（如8羟基脱氧鸟苷）从而产生DNA氧化损伤^[14]^。在AKR超家族中，AKR1C3是一种特别重要的酶，它可以代谢含2环以上的反式二氢二醇^[14]^。其他重要的酶还包括8-羟基鸟嘌呤DNA糖苷酶（8-oxoguanine DNA glycosylase, OGG1），它与碱基切除修复的氧化损伤修复途径有关^[15, 16]^。研究暴露于高浓度室内空气污染物，特别是暴露于高浓度PAHs的宣威人群，对于认识基因型与肺癌风险的关联具有重要价值。为了深入探索宣威肺癌风险的危险因素及发病机理，我们开展了两个基于人群的病例对照研究分别探讨了不同燃料种类和基因型差异与肺癌风险性的关系。

## 1 材料与方法

### 1.1 第一个基于人群的病例对照研究

第一个病例对照研究开展于1985年-1990年，其主要研究目的之一是探索烟煤种类与肺癌风险的关联。病例是于1985年11月-1990年2月在宣威的4家主要医院征集的新诊断肺癌患者，病例的合格条件是：①农民；②年龄18岁-85岁；③在诊断前居住在宣威至少1年以上。总共征集了500个肺癌病例，其中2例由于地址不正确而被剔除。在有效征集的498例肺癌病例中，303例仅经过X线胸片和临床诊断，195例（39%）还经痰液或支气管纤维镜、手术或活检穿刺等细胞学、病理学诊断。其中51例有组织学分类的病例中，39%是鳞状细胞癌，43%是腺癌，10%为未分化癌，8%为小细胞癌。

对照选取：在病例诊断4周内，采用分层随机抽样法在宣威的20个乡镇内随机选择乡（公社），然后在乡内随机选择行政村（大队），在行政村内随机抽取自然村（小队），最后在自然村随机选择一名性别和年龄（±2岁）与病例匹配的对照。由于乡镇间的人口数量差异比行政村和自然村大，本研究根据乡镇人口数占宣威全部乡镇总人口的比例设计了各乡镇的加权值（如A乡人口数占总人口比例为20%，加权值=0.2；B乡人口数占总人口比例为10%，加权值=0.1，抽取乡级样本时A乡被选择的机率是B乡的2倍），从而保证在每个乡镇里的对照研究对象被选择的机率是相似的。

问卷调查在研究对象的家中（19%病例和96%对照）或在医院完成（81%病例和4%对照）。问卷内容信息包括生活燃料使用、居住环境、做饭、室内外停留时间、吸烟、环境烟草暴露、医疗、家族恶性肿瘤患病史、饮食方式以及社会经济状况等。对照和病例的参与率分别达97%和100%。

在20世纪80年代，宣威90%以上的居民是农民。他们居住情况非常稳定，男性一般一生中都生活在同一村庄，而女性一般在结婚后迁居到其丈夫家里。即使这样，女性也一般都不会离开她们出生的乡镇。用煤家庭一般每年从离家最近的煤矿购买或采掘煤碳。一般来说，在一生中家庭年用煤量基本不变。累积燃煤量可通过年用煤量与燃煤年数的乘积得到。根据当地煤矿地质特征分析，本研究将宣威烟煤种类按煤的来源分为10个不同种类，分别按10个不同的乡镇命名。

### 1.2 第二个基于人群的病例对照研究

为了研究基因型的差异与肺癌风险的关联，1995年3月-1996年3月间又开展了一次以宣威居民为目标人群的病例对照研究。从宣威的4家主要医院和昆明的1家医院（经这5家医院诊断的肺癌患者包括了绝大部分同期被诊断的宣威肺癌病例）共征集了新发肺癌病例135例，其中参与本研究133例，参与率达98%。病例的征集条件包括：①经细胞学、病理学检查确诊为肺癌（105例，占78.9%）；或者②研究对象在1年内死亡（17例，12.8%）。对宣威肺癌病例的随访观察显示宣威肺癌病例的1年生存率为43.2%，因而本研究采用了1年内非其他原因死亡作为对仅有X胸片和临床体征病例的补充诊断方法。共有122例肺癌病例满足这些条件，其中包括24例非小细胞癌、3例小细胞癌和8例其他类型肺癌。

在征集的病例获得诊断后的2周内，通过户口登记系统随机在病例所居住的同一村庄选择一名年龄（±2岁）、性别和目前用来做饭和取暖的燃料种类都匹配的对照。对照的参与率为100%。

对每位参与本研究的研究对象都进行了标准问卷调查，内容包括人口统计学信息、吸烟史、家庭和个人医疗史、用煤量信息等。用煤量通过询问研究对象每年购买多少车拖拉机的煤来估计吨数，同时并询问用煤量是否有变化。烟煤的累积暴露量通过年用量与使用年数的乘积计算得到。

每个病例和对照均被采集一份口腔细胞样品，用于基因型分型分析。采集时用牙刷刷取口腔细胞，然后用生理盐水冲洗掉牙刷上的细胞，最后将口腔细胞放置在另一种含50%乙醇的盐溶液中于-20 ℃下保存。口腔细胞中的DNA提取方法参见文献^[17]^。*GSTM1*和*GSMTT1*基因型的分析采用了基于聚合酶链式反应（polymerase chain reaction, PCR）的方法^[18, 19]^。此方法在同一放大混合物中同时使用了GST特异性引物对和β-球蛋白的第3个引物对。GSTM1缺失或GSTT1缺失的特异性片段显示出空白基因型。β-球蛋白的特异性片段则用于对PCR是否足量的阳性控制。每个样品采样单盲法分析2次，对实验室来说样品的病例或对照的状态是未知的。

另外，还采集了每位研究对象的痰液样品，并用苯酚-三氯甲烷提取了痰样中的DNA。在122对病例对照中，成功地提取了119个病例和113名对照的DNA。基因型扫描采用ABI 7900HT系列检测系统的实时PCR法，详见SNP500的网站^[20]^。

### 1.3 统计学方法

在分析煤种、*GST*基因型与肺癌关联性时采用了条件*Logistic*回归分析估计了比数比（odds ratios, ORs）及其95%CI。在分析煤种与肺癌关联模式中，根据先前的研究结果，本研究以预计肺癌风险低的使用无烟煤和木柴的研究对象作为了参照。煤种模型中包括了使β参数值改变10%以上的可能混杂因子，或者以前的研究显示与肺癌风险有关联的因子。最终多因素*Logistic*回归模型包括了年龄、性别、教育程度、直系亲属肺癌患病史、在家中逗留的累积时间、肺部疾病史、吸烟及被动吸烟暴露史等。另外，还按性别进行了煤种与肺癌风险的分层回归分析。

GST基因型模型中调整的因子包括使用烟煤、吸烟的包年数（定义为每日吸烟包数与吸烟年数的乘积，假定1支香烟1 g烟草量）、慢性阻塞性肺部疾病（chronic obstructive pulmonary disease, COPD）和家庭成员肺癌史。AKR、OGG1基因型数据分析以最常用的基因型为参考，但AKR1C3例外，因为其最常见变型与ROS的产生有关联。数据分析采用了非条件*Logistic*回归模型以估计ORs和95%CIs。调整的因子有年龄、性别和烟草包年数。本研究还按性别和烟煤用量分层分析了AKR1C3、OGG1和GSTM1基因型与肺癌的关联性，调整了年龄、吸烟的包年数（仅男性）。运用了所观测的基因型频数和一个自由度的*χ*^2^检验，本研究针对对照进行了遗传平衡检验（*Hardy-Weinberg equilibrium* test）。

以上所有数据分析皆采用了SAS统计分析软件包（SAS Institute, Cary, NC）。

## 2 结果

### 2.1 煤种与肺癌风险

研究对象出生时的室内燃煤种类与肺癌的分析结果参见图 1。与燃用无烟煤和木柴相比，燃用烟煤的肺癌风险增高（OR=7.7, 95%CI: 4.5-13.3），其幅度视烟煤的种类不同有很大的变化。按其肺癌风险OR（95%CI）排序，分别为来宾24.8（12.4-49.6）、龙潭11.6（5.0-27.2）、宝山6.0（2.2-16.7）、龙场4.1（2.0-8.6）、羊场3.8（1.8-8.3）、文兴3.8（1.4-10.5）、倘塘2.8（1.2-6.6）、双河1.1（0.2-4.7）、杨柳0.7（0.2-3.1）、田坝0.6（0.2-1.9）等烟煤。异质性检验显示差异具有统计学意义（*P*=5.17×10^-10^）。

**1:**
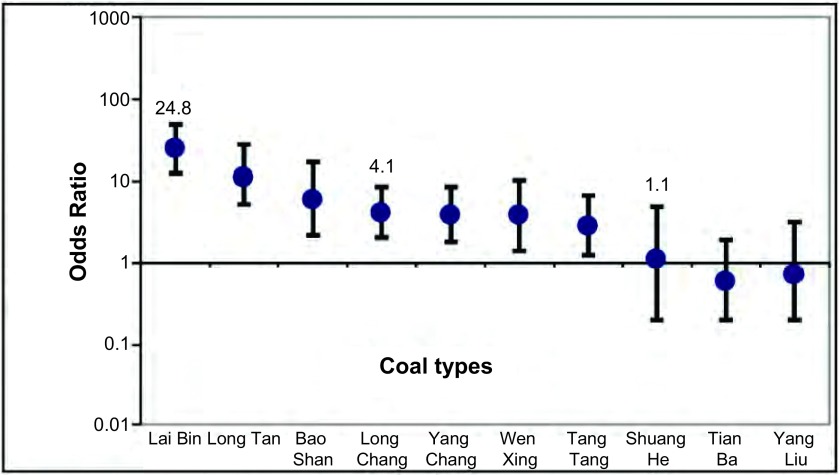
不同煤种的肺癌危险性 ORs of lung cancer in relation to coal type

按男女性别分层后的煤种与肺癌风险分析显示男性和女性的肺癌风险与煤种的关联相似（图 2）。燃用来宾烟煤对于男性和女性的肺癌风险与燃用无烟煤和木柴相比皆为最高，分别为OR=25.2（95%CI: 9.4-67.3）和26.4（95%CI: 9.6-72.6）；龙潭其次，分别为OR=14.8（95% CI: 4.2-51.5）和9.4（95%CI: 2.8-31.1）。与女性肺癌关联度大的煤种也与男性肺癌有很强关联（男性与女性OR的*Spearman*相关系数为0.89，*P*=0.000, 5）。另外，按吸烟与未吸烟分层分析显示肺癌风险在吸烟者与非吸烟者之间相似。本研究对煤种与肺癌风险关联性的分析结果进一步提供了家用不同的煤种的肺癌风险存在较大差异的依据。

**2:**
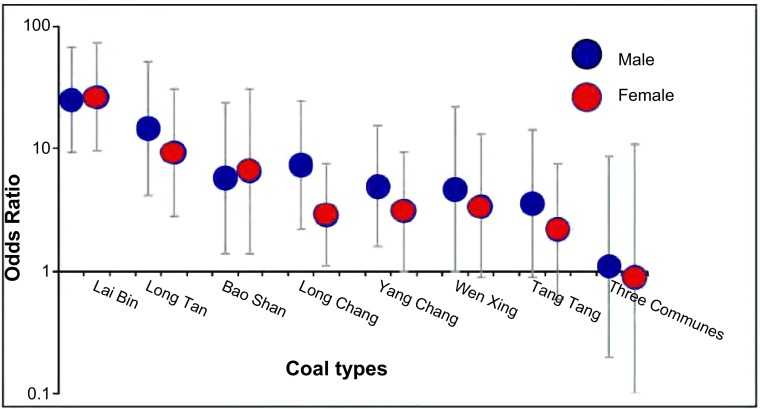
按性别分层的不同煤种的肺癌危险性 ORs of lung cancer in relation to coal type, stratified by gender

### 2.2 基因型与肺癌风险

图 3显示了3种基因型与肺癌风险的关联关系。对于*GSTM1*缺失基因型（病例组67.2% *vs*对照组50.8%）和AKR1C3（Ex1-70C > G）、OGG1（Ex6-315C > G）基因型，本研究发现肺癌风险明显增高，且具统计学意义[OR（95%CI）分别为2.3（1.3-4.2）、1.8（1.0-3.5）和1.9（1.1-3.3）]。

**3:**
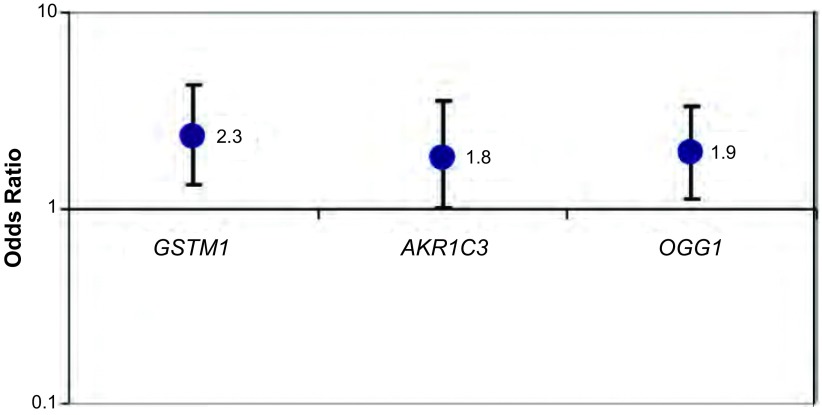
GSTM1缺失基因型和AKR1C3 (Ex1-70C > G)和OGG1 (Ex6-315C > G)基因型和肺癌风险 GSTM1-null, AKR1C3 (Ex1-70C > G), and OGG1 (Ex6-315C > G) genotype and lung cancer risk

对于家用烟煤累积用量小[ < 130 t（对照组的烟煤累积用量的均值）]，与基因型为GSTM1阳性的研究对象相比，具有GSTM1缺失基因型的女性肺癌风险OR为2.2（95%CI: 0.5-9.3），男性OR为1.5（95%CI: 0.6-3.7）；但对于家用烟煤累积用大（≥130 t）的研究对象，具有GSTM1缺失基因型的肺癌风险在女性中OR为4.9（95%CI: 1.3-18.2），男性OR为2.7（95%CI: 1.0-7.4）（表 1）。这提示无论男女，GSTM1基因型与燃用烟煤有联合效应。与GSTM1基因型不同的是，AKR1C3和OGG1基因型仅在燃用烟煤量很高的女性当中显示出明显的高肺癌风险[OR（95%CI）分别为12.9（2.2-107.8）和5.7（1.1-34.2）]。由于这两种基因型在BaP的代谢途径中发挥重要作用，这提示可能与女性在家里烧火做饭暴露于高浓度的PAHs有关。

**1:** 按性别与烟煤用量分层的*AKR1C3*、*OGG1*和*GSTM1*基因型与肺癌风险(ORs和95%CIs） *AKR1C3*, *OGG1*, and *GSTM1* genotype and lung cancer risk (ORs and 95%CIs), by sex and level of the lifetime exposure to smoky coal combustion

Genotype	Female			Male	
	Level of smoky coal use < 130 t	Level of smoky coal use≥130 t		Level of smoky coal use < 130 t	Level of smoky coal use≥130 t
GSTM1 null *vs* positive	2.2 (0.5-9.3)	4.9 (1.3-18.2)		1.5 (0.6-3.7)	2.7 (1.0-7.4)
AKR1C3 (Ex1-70C > G)					
GG *vs* CC+GC	1.00 (0.2-5.8)	12.9 (2.2-107.8)		1.5 (0.5-4.4)	0.9 (0.2-5.0)
OGG1 (Ex6-315C > G)					
GG+GC *vs* CC	1.3 (0.3-5.3)	5.7 (1.1-34.2)		1.1 (0.5-2.9)	2.0 (0.7-5.6)
ORs: odds ratios.					

## 3 讨论

宣威地区煤田成煤于晚二叠纪，但由于成煤物质、成煤环境以及后期成煤过程中地压、地温及变质时间等多种因素的差异，不同地理位置来源煤种的组分及其燃烧产物变化很大。煤的不完全燃烧可能产生许多挥发性或不挥发的可吸入性颗粒，如B(a)P、苯、甲醛等^[5]^，不同类型煤的使用对健康的危害主要取决于其组成成分。居民生活使用燃煤的种类不同，所产生的肺癌危险性有明显的差别，其中燃烟煤的肺癌风险大于无烟煤和木柴，暴露于来宾、龙潭地区烟煤的风险明显高与其他地区。在男性与女性或吸烟与非吸烟人群间相同煤种暴露的肺癌风险相似，不同煤种的肺癌风险相差较大。这些特点与宣威不同地区煤种燃烧产生的室内环境B(a)P浓度检测结果^[2-4]^和多次肺癌死亡率调查结果相吻合^[3, 4, 21-23]^，而且在宣威周边地区肺癌与PAHs暴露研究结果也显示不同位置来源的煤燃烧产生了不同种类或不同浓度的致癌物^[24-27]^。其次，本研究为基于农村人群的病例对照研究，人群的居住环境、生活燃料和燃煤方式相对稳定，并且远离汽车尾气、工业生产排放污染和职业暴露，与城市等其他地区研究相比，受到不可预测暴露的干扰相对较小，研究结果从煤种类型方面加强了宣威肺癌高发与燃煤污染的关联。

GSTM1、AKR1C3和OGG1等与致癌物质解毒、DNA氧化损伤和修复有关的酶均与宣威肺癌发病风险有关。GSTM1缺失基因型与肺癌风险增高有明显关联，GSTM1基因型缺失人群患肺癌的危险性是非缺失人群的2.3倍，而且男、女性肺癌风险均随着家庭生活燃煤量的增加而提高，特别是主要承担烧火做饭、日常家务而在室内逗留时间长、B(a)P等致癌物较高浓度暴露的女性患肺癌的风险更高。*AKR1C3*和*OGG1*基因型与肺癌的关联度相对较弱，仅在燃用烟煤量大于130 t的女性中显示出高肺癌风险，而在男性和燃煤量较低的女性人群中没有显示出明显的肺癌风险。以往一些研究表明宣威非吸烟女性肺癌与室内燃煤、B(a)P等暴露的风险大于男性^[2-4]^，女性亲属肺癌家族聚集性风险高于男性亲属^[4, 8]^，本研究在调整烟煤使用、肺癌家族史等因素后，女性对基因多态性的敏感仍高于男性，再次说明烟煤污染对肺癌的决定作用，宣威地区人群*GSTM1*、*AKR1C3*和*OGG1*基因型与烟煤用量存在联合效应。

Wacholder等^[28]^指出样本量较小的研究中，得出的关联关系出现假阳性结果的可能也较高。由于肺癌患病率以及突变基因型的频率在自然人群中分布较低，本研究无论从煤种、还是基因型与肺癌关系研究方面都存在样本量较小的缺陷。有些煤种因产地范围窄（局限在个别乡镇）或产量低或对肺癌的危险性小，被征集的病例数过少对研究的结果产生一些影响。研究地区为西部欠发达地区，医疗资源有限，当地被细胞学、组织学确诊的肺癌比例较低（约40%左右），诊断可靠性较低的病例又不便作为基因多态性研究对象，所以人群*GSTM1*、*AKR1C3*和*OGG1*基因型研究同样也受到样本量和样本代表性的制约。本研究的结果尚待更多（尤其是滇东同类地区）的研究结果的重现和支持。
